# Suspected Motor Problems and Low Preference for Active Play in Childhood Are Associated with Physical Inactivity and Low Fitness in Adolescence

**DOI:** 10.1371/journal.pone.0014554

**Published:** 2011-01-18

**Authors:** Marko T. Kantomaa, Jarno Purtsi, Anja M. Taanila, Jouko Remes, Helena Viholainen, Pauli Rintala, Timo Ahonen, Tuija H. Tammelin

**Affiliations:** 1 LIKES – Research Center for Sport and Health Sciences, Jyväskylä, Finland; 2 Finnish CP Association, Helsinki, Finland; 3 Institute of Health Sciences, University of Oulu, Oulu, Finland; 4 Unit of General Practice, University Hospital of Oulu, Oulu, Finland; 5 Finnish Institute of Occupational Health, Oulu, Finland; 6 Department of Education, University of Jyväskylä, Jyväskylä, Finland; 7 Department of Sport Sciences, University of Jyväskylä, Jyväskylä, Finland; 8 Department of Psychology, University of Jyväskylä, Jyväskylä, Finland; Pennington Biomedical Research Center, United States of America

## Abstract

**Background:**

This prospective longitudinal study investigates whether suspected motor problems and low preference for active play in childhood are associated with physical inactivity and low cardiorespiratory fitness in adolescence.

**Methodology/Principal Findings:**

The study sample consisted of the Northern Finland Birth Cohort 1986 (NFBC 1986) composed of 5,767 children whose parents responded to a postal inquiry concerning their children's motor skills at age 8 years and who themselves reported their physical activity at age 16 years. Cardiorespiratory fitness was measured with a cycle ergometer test at age 16 years. Odds ratios (OR) and their 95% confidence intervals (95% CI) for the level of physical activity and fitness were obtained from multinomial logistic regression and adjusted for socio-economic position and body mass index. Low preference for active play in childhood was associated with physical inactivity (boys: OR 3.31, 95% CI 2.42–4.53; girls: OR 1.79, 95% CI 1.36–2.36) and low cardiorespiratory fitness (boys: OR 1.87, 95% CI 1.27–2.74; girls: OR 1.52, 95% CI 1.09–2.11) in adolescence. Suspected gross (OR 2.16, 95% CI 1.33–3.49) and fine (OR 1.88, 95% CI 1.35–2.60) motor problems were associated with physical inactivity among boys. Children with suspected motor problems *and* low preference for active play tended to have an even higher risk of physical inactivity in adolescence.

**Conclusions/Significance:**

Low preference for active play in childhood was associated with physical inactivity and low cardiorespiratory fitness in adolescence. Furthermore, children with suspected motor problems *and* low preference for active play tended to have an even higher risk of physical inactivity in adolescence. Identification of children who do not prefer active play and who have motor problems may allow targeted interventions to support their motor learning and participation in active play and thereby promote their physical activity and fitness in later life.

## Introduction

Despite the known benefits of physical activity on health and well-being [1], recent evidence consistently demonstrates that a majority of adolescents do not meet current physical activity recommendations of at least 60 minutes per day of moderate or vigorous intensity activity at least five days per week [2]–[4]. At the same time, the level of cardiorespiratory and muscle fitness has decreased and body mass index has increased among adolescents and young adults [5]–[7]. According to epigenetic theories, behavioral changes in childhood are likely to launch children on to new developmental trajectories for the rest of their life spans [8]. Thus, motor proficiency and active play in childhood may be important determinants of subsequent physical activity and fitness, and hence serve as important tools for promoting health and well-being at all ages.

Previous cross-sectional studies suggest that motor proficiency is positively associated with physical activity in children [9]–[16] and adolescents [17], [18]. Furthermore, one longitudinal study observed that object control skills in childhood predicted physical activity level in adolescence, but locomotor skills were not associated with adolescent physical activity [19]. However, an intervention aimed at improving childhood motor skills did not impact physical activity level in long-term follow-up in Australian adolescents [20].

There is also evidence from cross-sectional studies that low motor competence may be associated with low physical fitness among children [21]–[23] and adolescents [21], [24], especially in terms of aerobic and anaerobic endurance, muscular strength and speed or agility [10], [21]–[25]. In addition, recent longitudinal studies reported that object control proficiency in childhood was associated with cardiorespiratory fitness in adolescence [26], [27], and that childhood motor skills were associated with cardiovascular endurance in early adolescence [28]. However, various methods have been used to measure physical fitness in previous studies [22], and therefore comparison of the results is difficult.

Much of young children's physical activity can be seen as playful in the sense that the activity is minimally constrained by adult demands [29]. It has been suggested that active play, defined as locomotor movements with a dimension of physical vigor in a playful context, may be one of the most important factors influencing human development [29]. These benefits include improved physical fitness, cognitive performance and motor development [29]. Despite the well-known benefits of active play on child development, no studies were found that investigated the association between childhood preference for active play and subsequent physical activity.

Since childhood motor proficiency and active play are closely related [23], together they may serve as important, modifiable determinants of subsequent physical activity and fitness. However, evidence from longitudinal population-based studies is scarce and the findings somewhat inconsistent, giving a relatively narrow picture of the phenomenon. The purpose of this prospective longitudinal study was to investigate the relationship between suspected motor problems and preference for active play in childhood and physical activity and cardiorespiratory fitness in adolescence. Additionally, we wanted to test the combined effects of suspected gross and fine motor problems and low preference for active play on subsequent physical activity and fitness. In this study, the term suspected motor problems will be used interchangeably with signs of low motor competence, low motor proficiency and movement difficulties, referring to possible presence of gross and/or fine motor problems. We hypothesized that suspected motor problems and low preference for active play in childhood are associated with physical inactivity and low cardiorespiratory fitness in adolescence.

## Materials and Methods

### Ethics statement

The Northern Finland Birth Cohort 1986 study conformed to the principles of the Declaration of Helsinki. The participants took part voluntarily and signed informed consent forms, which were also obtained from the children's parents. The Ethics Committee of the University Hospital of Oulu approved the research protocol.

### Participants

The study sample consisted of a prospective mother-child birth cohort, the Northern Finland Birth Cohort 1986 (NFBC 1986), which at the baseline was composed of 9432 infants who were born alive and whose expected date of birth was between July 1, 1985, and June 30, 1986, in the two northernmost provinces of Finland, Oulu and Lapland [30].

The data collection started during the mothers' pregnancy, and the follow-up surveys were carried out when the children were age 7–8 years (1992–1994) and 15–16 years (2001–2002) (hereafter referred to as ‘16 years’). When the children were 7–8 years, parents were sent a postal inquiry including questions about the children's growth and health, behavior and performance and family conditions (response rate 90%). At the age of 16 years, adolescents were sent a postal questionnaire including questions about health and well-being (response rate 80%, N = 7344). Parents were also sent a postal inquiry including questions about family conditions (response rate 76%, N = 6985). At the age of 16 years, cohort members also participated in individual health examinations (participation rate 74%, N = 6798) including measurement of cardiorespiratory fitness (N = 5375). The present analyses included those 5767 children whose motor skills and preference for active play at age 8 years and physical activity at age 16 years were reported.

### Motor skills and preference for active play at age 8 years

Gross and fine motor skills at the age of 8 years were measured with the parents' questionnaire. Parents were asked the following questions regarding gross motor skills: ‘Does your child bump into something or fall down often?’ (response alternatives: 1) yes, 2) no and 3) cannot say); ‘Can your child usually catch the ball in the game?’ (1) mostly, 2) sometimes and 3) hardly ever) and ‘Can your child a) ride a two-wheel bike or b) skate?’ (1) yes, 2) no and 3) not attempted). A child was defined as having *suspected gross motor problems* if he or she had frequent bumps or falls, hardly ever succeeded in catching a ball in a game or was unable to ride a two-wheel bike or skate.

Fine motor skills were investigated with the following questions: ‘Is your child's pencil use awkward?’ (1) yes, 2) no and 3) cannot say); ‘Can your child tie his or her shoelaces?’ (1) yes, 2) no and 3) not attempted) and ‘Can your child use scissors?’ (1) yes and 2) no). A child was classified as having *suspected problems with fine motor skills* if parents thought that their child's pencil use was awkward, the child could not tie his or her shoelaces or the child could not use scissors.

A child's preference for active play was evaluated by asking parents the following question: ‘Does your child like to participate in active play?’ (response alternatives: 1) often, 2) sometimes and 3) hardly ever). Those children falling into category 3 (hardly ever) were classified as having *low preference for active play*. In this study the term ‘active play’ refers to ‘physical activity play’ or ‘locomotor play’, the terms commonly used in child play and development research [8], [29].

For a more accurate examination of suspected childhood motor problems and low preference for active play, we formed eight subgroups according to different combinations of these problems: G1 (no problems), G2 (suspected gross motor problems [GMP] only), G3 (suspected fine motor problems [FMP] only), G4 (low preference for active play [LPAP] only), G5 (suspected gross and fine motor problems [GMP & FMP]), G6 (suspected gross motor problems and low preference for active play [GMP & LPAP]), G7 (suspected fine motor problems and low preference for active play [FMP & LPAP]) and G8 (suspected gross and fine motor problems and low preference for active play [GMP & FMP & LPAP]).

### Physical activity at age 16 years

Physical activity level was defined as metabolic equivalent-hours per week (MET hours per week) based on the intensity and volume of physical activity engaged in outside school hours, including commuting to and from school [3], and was divided into quintiles. The amount of physical activity outside school hours was evaluated separately for moderate-to-vigorous physical activity and light physical activity by asking, ‘How many hours a week altogether do you participate in a) brisk and b) light physical activity outside school hours?’ In the questionnaire, the term brisk was defined as physical activity causing at least some sweating and getting out of breath (here referred to as moderate-to-vigorous intensity physical activity), and the term light as physical activity causing no sweating or shortage of breath. In addition, the adolescents were asked about their daily time spent in physically active commute to and from school. The response alternatives (not at all, less than 20 min, 20–39 min, 40–59 min, and at least 1 hour per day) were multiplied by five (five school days a week) to correspond to 0, 1, 2.5, 3.75 and 5 hours per week. A MET intensity value of 3 METs was used for light physical activity, 5 METs for brisk physical activity and 4 METs for commuting physical activity in calculations [31]. MET hours were divided into gender-specific quintiles: 1) active (two highest quintiles), 2) moderately active (third and fourth quintiles) and 3) inactive (lowest quintile).

The test-retest reliability of the individual physical activity questions used here has been reported to be fairly good among Finnish adolescents age 15–16 years [3]. The intraclass correlation coefficients for physical activity level described as quintile categories of MET hours per week was 0.70 (95% confidence interval 0.58–0.80), and the proportion of subjects who were classified in exactly the same category or next to the same category in two different tests was 86%.

### Cardiorespiratory fitness at age 16 years

Cardiorespiratory fitness was measured in connection with an individual health examination with a submaximal cycle ergometer test and expressed as peak oxygen uptake (VO_2peak_) in ml·kg^−1^·min^−1^. Subjects were categorized into gender-specific quintiles of fitness: 1) high (two highest quintiles), 2) average (third and fourth quintiles) and 3) low (lowest quintile). The exercise test protocol included two incremental work stages of 4 min each on a bicycle ergometer (model 818E, Monark, Sweden). Peak oxygen uptake (VO_2peak_ in ml·kg^−1^·min^−1^) was calculated based on the heart rate response during submaximal work stages. The method has been validated against directly measured VO_2peak_ during the maximal exercise test and has been previously described in detail [3].

### Confounding factors

Information about parental socio-economic position was obtained from the parents' questionnaire in the autumn of the child's first school year, at the age of 7 years. Socio-economic position was based on the mother's and father's occupations. Parental socio-economic position was classified into eight groups: professionals (fathers 43%, mothers 36%) included 1) employers and own-account workers and 2) upper-level white-collar workers, whereas non-professionals (fathers 57%, mothers 64%) included 3) lower-level white-collar workers, 4) blue-collar workers, 5) farmers, 6) students, 7) pensioners and 8) others [32].

Parents reported in a questionnaire their children's body weight and height at the age of 7 years. At the age of 16 years, the children self-reported their body weight and height in the postal inquiry, and they were measured in the health examination. Self-reported body weight and height were used for those who failed to attend the health examination. Body mass index (BMI) was calculated as weight divided by the square of the height (kg/m^2^). Obesity was defined using the International Obesity Task Force (IOTF) age-specific cut-off points for BMI [33]. Participants were classified into five groups according their BMI from 7 to 16 years: 1) normal weight (normal weight at age 7 and 16 years, 80%), 2) overweight (overweight at age 7 and 16 years, 4%), 3) obesity (obese at age 7 and 16 years, 1%), 4) weight gain (normal weight or overweight at age 7 years but overweight or obese at age 16 years, 6%) and 5) weight loss (obese or overweight at age 7 years but overweight or normal weight at age 16 years, 9%).

### Statistical analyses

The basic analyses included frequency counts and relative distributions. Bivariate associations were tested separately for boys and girls with cross-tabulation with chi-square tests and multinomial logistic regression. Multivariable analyses were also performed separately for boys and girls using multiple multinomial logistic regression.

First, childhood suspected gross and fine motor problems and low preference for active play associated with adolescent physical inactivity and low fitness were tested separately. Second, to get more detailed information about these associations, we examined different combinations of childhood suspected motor problems and low preference for active play (subgroups G1–G8) in association with adolescent physical inactivity and low cardiorespiratory fitness.

The results of the regression analyses are presented with odds ratios (OR) and 95% confidence intervals (95% CI). In the multivariable models, the variables were adjusted for parental socio-economic position at age 7 years and for change in BMI from age 7 to 16 years. The data were analyzed using SPSS Software, version 16.0.

## Results

**Figure 1:**
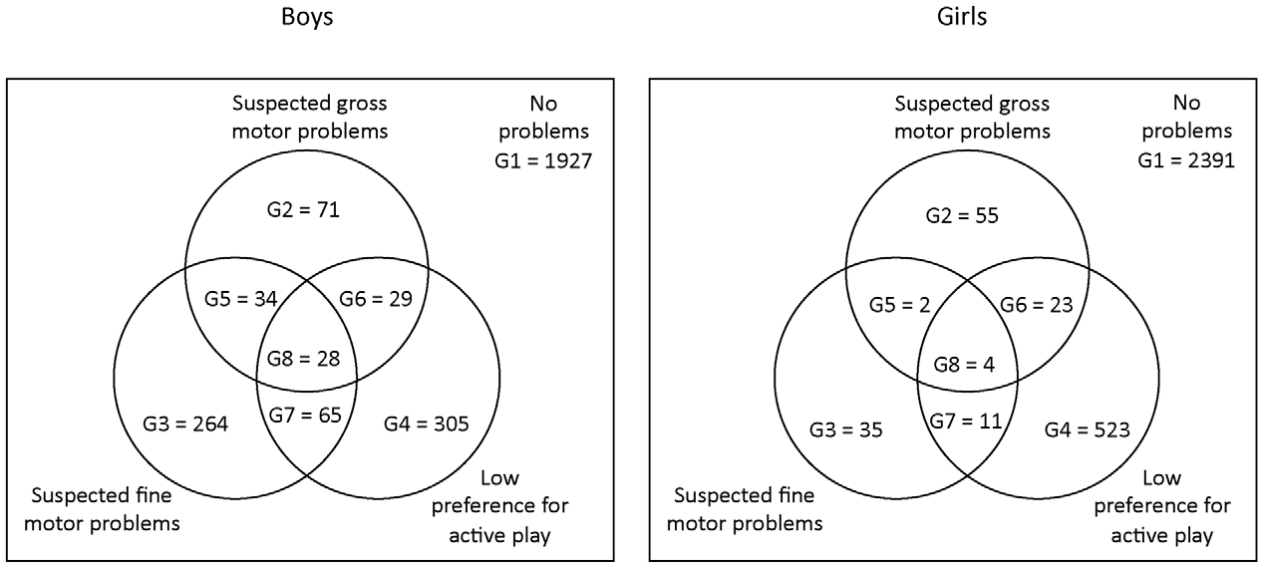
The number of boys (N = 2723) and girls (N = 3044) belonging to each subgroup according to suspected gross and fine motor problems and low preference for active play at age 8 years. Areas are non-proportional.

Table 1 displays the sample characteristics measured at age 8 and 16 years for the study sample. At age 8 years, boys had more suspected gross (p<0.001) and fine (p<0.001) motor problems compared with girls. Girls more often reported low preference for active play than boys (p = 0.006). The number of children in each subgroup of different combinations of suspected motor problems and low preference for active play (G1–G8) are presented in Figure 1.

**Table 1:** Sample characteristics of boys and girls in the Northern Finland Birth Cohort 1986.

	Boys		Girls	
	N	%	N	%
At age 8 years				
Suspected gross motor problems				
Yes	162	5.9	84	2.8
No	2561	94.1	2960	97.2
Suspected fine motor problems				
Yes	391	14.4	52	1.7
No	2332	85.6	2992	98.3
Low preference for active play^a^				
Yes	427	15.7	561	18.4
No	2295	84.3	2483	81.6
At age 16 years				
Physical activity level^b^				
Active	1146	42.1	1261	41.4
Moderately active	1068	39.2	1162	38.2
Inactive	509	18.7	621	20.4
Cardiorespiratory fitness level^c^				
High	855	39.7	884	40.0
Average	869	40.4	886	40.1
Low	428	19.9	439	19.9

a Low preference for active play was defined as parents reporting that children liked to participate in active play ‘hardly ever’.

b Metabolic equivalent-hours based on the intensity and volume of physical activity divided into gender-specific quintiles: 1) active (two highest quintiles), 2) moderately active (third and fourth quintiles) and 3) inactive (lowest quintile).

c Peak oxygen uptake (VO_2peak_) in ml·kg^−1^·min^−1^ divided into gender-specific quintiles: 1) high (two highest quintiles), 2) average (third and fourth quintiles) and 3) low (lowest quintile).

Boys were physically more active (p<0.001), and they had higher cardiorespiratory fitness (p<0.001) than girls at the age of 16 years. The mean MET hours per week were 33.3 (SD 17.8) among boys and 28.8 (SD 15.4) among girls. The mean peak oxygen consumption was 48.7 (SD 8.9) ml·kg^−1^·min^−1^ among boys and 34.2 (SD 5.8) ml·kg^−1^·min^−1^ among girls.

The unadjusted analyses for the associations between suspected motor problems and low preference for active play in childhood, and physical activity and cardiorespiratory fitness in adolescence are presented in Table 2. Since there were no significant differences between the unadjusted and the adjusted (for parental socio-economic position and change in BMI) results, we present only the adjusted results in the following paragraphs.

**Table 2:** The level of physical activity and cardiorespiratory fitness at age 16 years by suspected motor problems and low preference for active play at age 8 years. (%).

	Physical activity level^a^(Boys N = 2723, Girls N = 3044)			Cardiorespiratory fitness level^b^(Boys N = 2152, Girls N = 2209)		
	Active	Moderatelyactive	Inactive	High	Average	Low
Boys						
Suspected gross motor problems						
Yes	27.1	42.0	30.9	29.3	38.8	31.9
No	43.0	39.1	17.9	40.3	40.5	19.2
P-value^c^	<0.001			0.002		
Suspected fine motor problems						
Yes	31.5	43.0	25.5	36.3	41.4	22.3
No	43.9	38.6	17.5	40.3	40.2	19.5
P-value^c^	<0.001			0.320		
Low preference for active play^d^						
Yes	27.4	42.4	30.2	27.0	45.4	27.6
No	44.9	38.6	16.5	41.9	39.5	18.6
P-value^c^	<0.001			<0.001		
Girls						
Suspected gross motor problems						
Yes	27.4	47.6	25.0	29.0	45.2	25.8
No	41.8	37.9	20.3	40.3	40.0	19.7
P-value^c^	0.030			0.177		
Suspected fine motor problems						
Yes	32.7	36.5	30.8	40.0	42.9	17.1
No	41.6	38.2	20.2	40.0	40.1	19.9
P-value^c^	0.150			0.905		
Low preference for active play^d^						
Yes	35.1	40.3	24.6	35.4	39.5	25.1
No	42.9	37.7	19.4	41.1	40.2	18.7
P-value^c^	0.001			0.010		

a Metabolic equivalent-hours based on the intensity and volume of physical activity divided into gender-specific quintiles: 1) active (two highest quintiles), 2) moderately active (third and fourth quintiles) and 3) inactive (lowest quintile).

b Peak oxygen uptake (VO_2peak_) in ml·kg^−1^·min^−1^ divided into gender-specific quintiles: 1) high (two highest quintiles), 2) average (third and fourth quintiles) and 3) low (lowest quintile).

c Pearson's chi-square test.

d Low preference for active play was defined as parents reporting that children liked to participate in active play ‘hardly ever’.

Low preference for active play at age 8 years was associated with physical inactivity (boys: OR 3.31, 95% CI 2.42–4.53; girls: OR 1.79, 95% CI 1.36–2.36) and low cardiorespiratory fitness (boys: OR 1.87, 95% CI 1.27–2.74; girls: OR 1.52, 95% CI 1.09–2.11) at age 16 years (Table 3). Additionally, suspected gross (OR 2.16, 95% CI 1.33–3.49) and fine (OR 1.88, 95% CI 1.35–2.60) motor problems at age 8 years were associated with physical inactivity at age 16 years among boys, but not among girls. However, neither suspected gross nor fine motor problems in childhood were associated with cardiorespiratory fitness in adolescence (Table 3).

**Table 3:** Multinomial regression of physical activity and cardiorespiratory fitness at age 16 years on suspected motor problems and low preference for active play at age 8 years.

	Physical activity^a^(Boys N = 2203, Girls N = 2389)		Cardiorespiratory fitness^b^(Boys N = 1770, Girls N = 1776)	
	Moderately active vs. Active	Inactive vs. Active	Average vs. High	Low vs. High
	AdjustedOR (95% CI)^c^	AdjustedOR (95% CI)^c^	AdjustedOR (95% CI)^c^	AdjustedOR (95% CI)^c^
Boys				
Suspected gross motor problems				
Yes vs. No	1.60 (1.04–2.45)	2.16 (1.33–3.49)	1.10 (0.67–1.81)	1.59 (0.90–2.79)
Suspected fine motor problems				
Yes vs. No	1.41 (1.06–1.86)	1.88 (1.35–2.60)	1.03 (0.76–1.41)	1.07 (0.73–1.58)
Low preference for active play^d^				
Yes vs. No	1.97 (1.49–2.61)	3.31 (2.42–4.53)	1.79 (1.29–2.46)	1.87 (1.27–2.74)
Girls				
Suspected gross motor problems				
Yes vs. No	1.59 (0.87–2.93)	1.82 (0.91–3.62)	1.57 (0.76–3.26)	1.47 (0.63–3.43)
Suspected fine motor problems				
Yes vs. No	1.00 (0.45–2.20)	1.86 (0.84–4.14)	1.15 (0.51–2.60)	0.65 (0.20–2.09)
Low preference for active play^d^				
Yes vs. No	1.34 (1.06–1.71)	1.79 (1.36–2.36)	1.12 (0.84–1.48)	1.52 (1.09–2.11)

a Metabolic equivalent-hours based on the intensity and volume of physical activity divided into gender-specific quintiles: 1) active (two highest quintiles), 2) moderately active (third and fourth quintiles) and 3) inactive (lowest quintile).

b Peak oxygen uptake (VO_2peak_) in ml·kg^−1^·min^−1^ divided into gender-specific quintiles: 1) high (two highest quintiles), 2) average (third and fourth quintiles) and 3) low (lowest quintile).

c Adjusted for mother's and father's socio-economic positions when the children were 7 years old and for change in body mass index from 7 to 16 years. OR, odds ratio; 95% CI, 95% confidence interval.

d Low preference for active play was defined as parents reporting that children liked to participate in active play ‘hardly ever’.

Suspected gross motor problems *together* with low preference for active play (G6) were associated with physical inactivity among girls (OR 4.94, 95% CI 1.22–19.97) and low cardiorespiratory fitness among boys (OR 6.05, 95% CI 1.16–31.53) (Table 4). On the other hand, suspected fine motor problems *together* with low preference for active play (G7) were associated with physical inactivity among boys (OR 6.54, 95% CI 3.03–14.12). Finally, a combination of *all* of the childhood problems (G8) was associated with adolescent physical inactivity among boys (OR 4.59, 95% CI 1.47–14.27).

**Table 4:** Multinomial regression of physical inactivity and low level of cardiorespiratory fitness at age 16 years on different combinations of suspected gross motor problems (GMP), fine motor problems (FMP) and low preference for active play (LPAP) at age 8 years.

	Physical activity^a^		Cardiorespiratory fitness^b^	
	Boys (N = 2203)	Girls (N = 2389)	Boys (N = 1770)	Girls (N = 1776)
	Inactive vs. Active	Inactive vs. Active	Low vs. High	Low vs. High
	AdjustedOR (95% CI)^c^	AdjustedOR (95% CI)^c^	AdjustedOR (95% CI)^c^	AdjustedOR (95% CI)^c^
Types of suspected motor problems				
G1. No problems	1.00	1.00	1.00	1.00
G2. GMP only	2.52 (1.25–5.05)	1.43 (0.61–3.36)	1.37 (0.61–3.10)	1.66 (0.64–4.34)
G3. FMP only	1.58 (1.05–2.37)	2.29 (0.85–6.20)	1.11 (0.70–1.77)	0.45 (0.10–2.12)
G4. LPAP only	3.34 (2.31–4.82)	1.74 (1.31–2.32)	1.82 (1.17–2.83)	1.49 (1.07–2.08)
G5. GMP & FMP	3.24 (1.03–10.20)	N/A	1.02 (0.33–3.16)	N/A
G6. GMP & LPAP	1.92 (0.62–5.97)	4.94 (1.22–19.97)	6.05 (1.16–31.53)	1.90 (0.26–13.82)
G7. FMP & LPAP	6.54 (3.03–14.12)	1.62 (0.36–7.42)	1.28 (0.53–3.13)	3.70 (0.32–42.16)
G8. GMP & FMP & LPAP	4.59 (1.47–14.27)	N/A	6.27 (0.65–60.59)	N/A

a Metabolic equivalent-hours based on the intensity and volume of physical activity divided into gender-specific quintiles: 1) active (two highest quintiles), 2) moderately active (third and fourth quintiles) and 3) inactive (lowest quintile).

b Peak oxygen uptake (VO_2peak_) in ml·kg^−1^·min^−1^ divided into gender-specific quintiles: 1) high (two highest quintiles), 2) average (third and fourth quintiles) and 3) low (lowest quintile).

c Adjusted for mother's and father's socio-economic position when the children were 7 years old and for change in body mass index from 7 to 16 years. OR, odds ratio; 95% CI, 95% confidence interval. Note: N/A  =  not available.

## Discussion

Our results suggest that low preference for active play in childhood is associated with physical inactivity and low cardiorespiratory fitness in adolescence. Additionally, children with suspected motor problems *together* with low preference for active play tend to be an even higher risk for physical inactivity in adolescence.

To the authors' knowledge, this is the first study investigating the influence of suspected childhood gross and fine motor problems and low preference for active play on physical activity and cardiorespiratory fitness in adolescence. However, one longitudinal study has reported that object control skills in childhood predict physical activity in adolescence, independent of gender [19]. Interestingly, in our study the association between suspected childhood motor problems and adolescent physical inactivity was more consistent among boys than girls. This could be partly explained by significant differences in the occurrence of suspected motor problems according to gender in the present study sample. On the other hand, it may be that differences in types of physical activities typical of boys and girls may improve diverse motor skills, which, in turn, may have various impacts on subsequent physical activity.

The present study adds to previous findings by showing that children with suspected motor problems *and* low preference for active play tended to have an even higher risk of physical inactivity in adolescence than children with *either* suspected motor problems *or* low preference for active play. Hands and Larkin [23] have suggested that children with low motor competence are less active than children with high motor competence, and consequently, their development of physical fitness, as well as skills, is compromised. According to Hands and Larkin [23], this leads to continuous negative interaction among low motor competence, physical inactivity and low physical fitness. Our results support this hypothesis, additionally indicating a close and developmentally significant relationship between motor learning and active play in childhood. This relatively unique finding may be crucial for future physical activity and health promotion practices.

Recently, Barnett et al. [26] reported that motor proficiency in childhood predicted cardiorespiratory fitness in adolescence. Inconsistently, our results showed no association between suspected motor problems in childhood and cardiorespiratory fitness in adolescence. It is possible that these inconsistencies are due to the fact that we studied suspected motor problems based on parent report, instead of objectively measured, more accurately defined motor problems. However, these inconsistencies may also be due to various measures of physical fitness in previous studies, many of which require coordination and motor planning [22]. These test items might be difficult to perform for young people with motor problems, and may therefore contribute to poor test outcomes [23]. The cycle ergometer test used to measure cardiorespiratory fitness in the present study is likely to be a more objective measure of physical fitness in this respect.

The present results show a consistent association between low preference for active play in childhood and a low level of cardiorespiratory fitness in adolescence. Previous studies have identified associations between active play and physical fitness, suggesting that active play can serve immediate and deferred functions for endurance and strength [29]. This may be partly explained by positive changes in muscle fibers and improved skill and economy of movement followed by active play in childhood [29]. It is also possible that active play has an influence on a child's enjoyment and motivation to participate in subsequent physical activity, which, in turn, is an important determinant of physical fitness in adolescence [1]. Although further research is needed, our results indicate that low preference for active play in childhood could serve as a simple indicator of later risk of physical inactivity and low fitness.

The rising levels of physical inactivity and epidemic of obesity reflect the profound cultural and environmental changes in society [34]. Since children, especially, are responsive to environmental and cultural changes, and adjust their behaviors in response to such changes [35], [36], motor learning and active play in childhood may provide an important opportunity for promotion of physical activity and fitness. The possible benefit of motor learning and active play, relative to other, adult-directed strategies, is that behaviors generated in the context of play can be more innovative than adult tuition, affording opportunities for subsequent practice of newly developed behaviors and strategies [37], [38]. From the public health point of view, promotion of health-enhancing behaviors and strategies through motor learning and active play in childhood is probably less costly and risky than in later life, and, therefore, more likely to spread through the population.

The following limitations need to be considered when interpreting the findings of this study. In the present study we measured suspected motor problems based on parental report rather than more accurately defined motor problems based on objective measurement, and could therefore be subject to unintentional recall bias, inaccuracy as well as potential deliberate reporting bias by the parents [39]. In addition, limited number of questions may give a somewhat narrow picture of the phenomenon. Furthermore, the questions used to measure suspected motor problems and preference for active play have not been validated in Finnish children. Although the prevalence of suspected motor problems in this study sample was relative to the prevalence of motor problems reported in previous studies [40], it is possible that the gender difference in the occurrence of these problems in the present study is partly explained by the weaknesses of parent perceptions. However, the gender difference in active play have been observed in previous studies, with males, more than females, engaging in active play more frequently and at higher levels of intensity [29]. Furthermore, the present study relied on self-reporting of adolescent physical activity, which might result in measurement errors and social desirability bias. For young people, errors in recall of physical activity are also likely to be greater than for adults [41], [42].

Our study has several strengths, including the large, unselected population sample, which provided a good opportunity to study active play and motor skills predicting subsequent physical activity and fitness. Furthermore, the prospective longitudinal study setting allows some conclusions about the direction of causality. Participation rates were high, 90% at age 8 years and 80% (questionnaire) and 74% (clinical examination) at age 16 years. Additionally, cardiorespiratory fitness was measured objectively with a cycle ergometer test, providing most likely reliable and comparable results for adolescents with and without motor problems.

It would be useful to investigate these associations in more contemporary cohorts and within different socio-cultural settings. Future research into the associations between motor problems and active play in childhood and physical activity in later life using objective measures would be particularly useful, as this may help identify the mechanisms and mediating factors that explain these associations. Also gender differences in motor problems and active play as such and in association with physical activity warrant further examination. Better identification of mediating and moderating variables would be especially beneficial for physical activity interventions, which could be targeted at improving these factors. It is also possible that the association between motor problems and subsequent physical activity varies according to the form of physical activity, for example, between organized and non-organized physical activity [18] or between different types of physical activity.

Our results suggest that low preference for active play in childhood may be an important predictor of physical inactivity and low cardiorespiratory fitness in adolescence. Furthermore, suspected childhood motor problems *together* with low preference for active play may be even stronger predictors of adolescent physical inactivity. Identifying children who do not prefer active play and who have motor problems might help target interventions to support children's participation in active play and motor learning with the aim of promoting physical activity and fitness later in life.
